# Temporary Mechanical Circulatory Support in Cardiogenic Shock Patients after Cardiac Procedures: Selection Algorithm and Weaning Strategies

**DOI:** 10.3390/life13102045

**Published:** 2023-10-12

**Authors:** Gaik Nersesian, Sascha Ott, Alexander Fardman, Pia Lanmueller, Daniel Lewin, Alexander Bernhardt, Fabian Emrich, Gloria Faerber, Gábor Szabó, Mehmet Oezkur, Bernd Panholzer, Sebastian V. Rojas, Diyar Saeed, Bastian Schmack, Gregor Warnecke, Daniel Zimpfer, Herko Grubitzsch, Volkmar Falk, Evgenij Potapov

**Affiliations:** 1 Department of Cardiothoracic and Vascular Surgery, Deutsches Herzzentrum der Charité (DHZC), Augustenburger Platz 1, 13353 Berlin, Germany; 2 German Center for Cardiovascular Research (DZHK), Partner Site Berlin, 10785 Berlin, Germany; 3 Department of Cardiac Anesthesiology and Intensive Care Medicine, Deutsches Herzzentrum der Charité (DHZC), 13353 Berlin, Germany; 4 Leviev Heart Center, Sheba Medical Center, Ramat-Gan, Israel Affiliated to the Sackler School of Medicine, Tel Aviv University, Tel Aviv 6997801, Israel; 5 Department of Cardiovascular Surgery, University Heart and Vascular Center Hamburg, 20251 Hamburg, Germany; 6 Department of Cardiac Surgery, Goethe University Hospital, 60590 Frankfurt, Germany; 7 Department of Cardiothoracic Surgery, Jena University Hospital, Friedrich-Schiller-University Jena, 07747 Jena, Germany; 8 Department of Cardiac Surgery, Middle German Heart Centre, University Hospital Halle (Saale), Martin-Luther University Halle-Wittenberg, 06120 Halle (Saale), Germany; 9 Department of Cardiovascular Surgery, University Hospital Mainz, 55131 Mainz, Germany; 10 Department of Cardiovascular Surgery, University Hospital Schleswig-Holstein, Campus Kiel, 24105 Kiel, Germany; 11 Clinic for Thoracic and Cardiovascular Surgery, Heart and Diabetes Center NRW, Ruhr-University Bochum, 32545 Bad Oeynhausen, Germany; 12 Department for Cardiac Surgery, Heart Center Niederrhein, 47805 Krefeld, Germany; 13 Department of Thoracic and Cardiovascular Surgery, West German Heart and Vascular Center, University of Duisburg-Essen, 45147 Essen, Germany; 14 Department of Cardiac Surgery, University Hospital Heidelberg, 69120 Heidelberg, Germany; 15 Department of Cardiothoracic Surgery, Medical University of Vienna, 1090 Vienna, Austria; 16 Charité—Universitätsmedizin Berlin, Corporate Member of Freie Universität Berlin, Humboldt-Universität zu Berlin, Berlin Institute of Health, 10178 Berlin, Germany; 17 Department of Health Sciences and Technology, ETH Zurich, 8092 Zurich, Switzerland

**Keywords:** postcardiotomy cardiogenic shock, mechanical circulatory support, extracorporeal life support, microaxial flow pump, Impella

## Abstract

Mechanical circulatory support has proven effective in managing postcardiotomy cardiogenic shock by stabilizing patients’ hemodynamics and ensuring adequate organ perfusion. Among the available device modalities, the combination of extracorporeal life support and a microaxial flow pump for left ventricular unloading has emerged as a valuable tool in the surgical armamentarium. In this publication, we provide recommendations for the application and weaning of temporary mechanical circulatory support in cardiogenic shock patients, derived from a consensus among leading cardiac centers in German-speaking countries.

## 1. Introduction

Cardiogenic shock (CS) in patients following cardiac surgery (postcardiotomy cardiogenic shock, PCCS) is a highly relevant but complex diagnosis. CS after heart surgery refers to a critical hemodynamic state of patients where weaning from cardiopulmonary bypass (CPB) is not possible or a persistent low cardiac output syndrome occurs during or after the cardiac procedure [1]. Although the incidence of PCCS is around 1–5%, it is associated with a considerable hospital mortality rate of over 60% [1,2,3].

The term “postcardiotomy” does not accurately encompass the range of cardiac procedures that can result in CS). While PCCS is most frequently observed after standard cardiac procedures such as coronary artery bypass grafting or heart valve surgery, it can also occur after endovascular procedures like transcatheter aortic valve implantation or interventions on the thoracic aorta. Despite the fact that these procedures do not involve opening of heart chambers, they are still broadly classified as PCCS [4]. Additionally, acute graft failure following a heart transplantation or right heart failure after left ventricular assist device (LVAD) implantation are also often described as special forms of PCCS [5]. In our opinion, a more appropriate term for the pathological condition this publication focuses on would be “low cardiac output syndrome after cardiac surgery”. Nevertheless, we will continue to use the conventional term PCCS to comply with common terminology [4].

**Figure 1:**
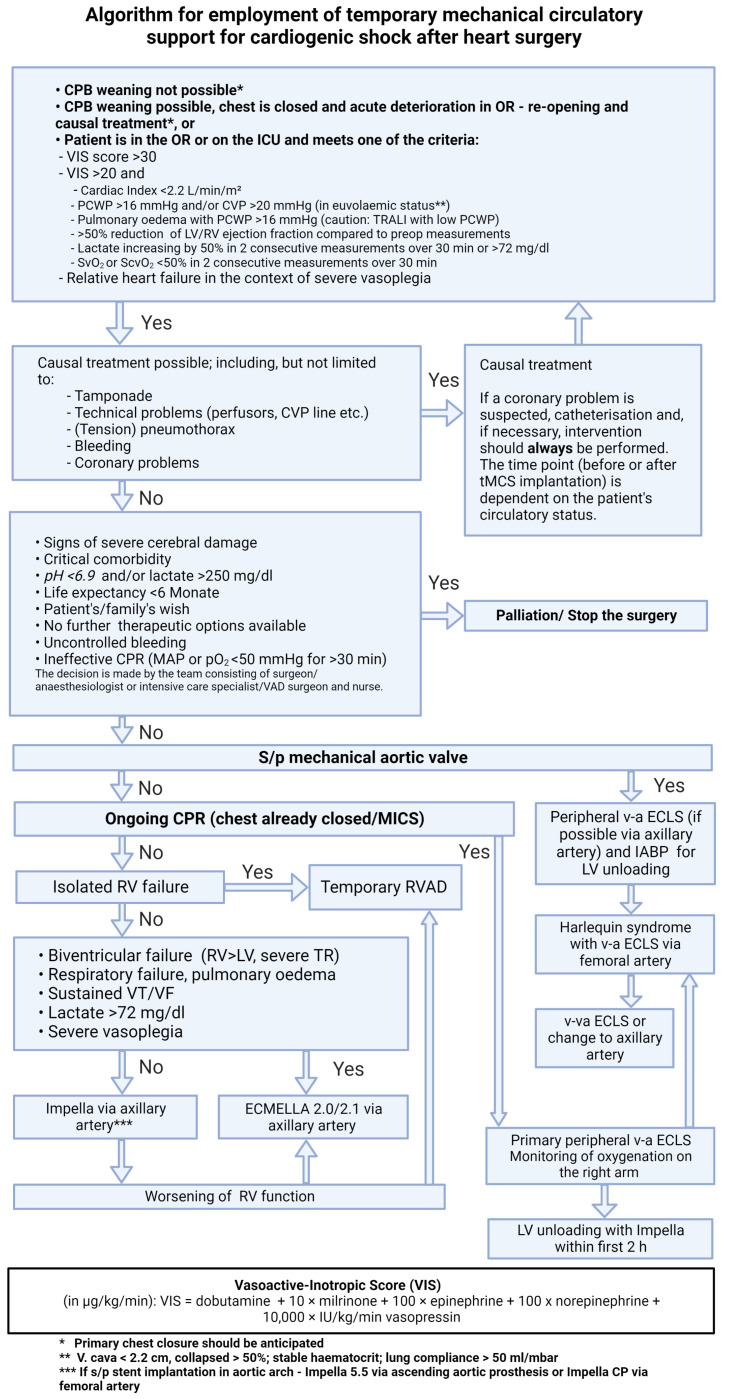
Algorithm for employment of temporary mechanical circulatory support for cardiogenic shock after heart surgery. Abbreviations: CPB—cardiopulmonary bypass; OR—operating room; ICU—intensive care unit; VIS—vasoactive inotropic score; PCWP—pulmonary capillary wedge pressure; TRALI—transfusion-related acute lung injury; CVP—central venous pressure; CPR—cardiopulmonary resuscitation; MAP—mean arterial pressure; s/p—status post; MICS—minimally-invasive cardiac surgery; RVAD—right ventricular assist device; v-a ECLS—veno-arterial extracorporeal life support; IABP—intraaortic balloon pump; L(R)V—left (right) ventricle.

In this article, we present an algorithm for the therapy allocation of patients with PCCS (Figure 1). This standardized approach represents a consensus among experts in the field of mechanical circulatory support (MCS) from 16 cardiac centers from German-speaking countries, and is based on the latest knowledge in this field [6].

## 2. General Principles

Temporary MCS devices play a key role in the therapy of PCCS. While a wide range of different temporary MCS systems and combinations thereof are available, no single therapy approach has yet established itself as the gold standard [6]. Nevertheless, for patients who develop PCCS during or after heart surgery, the early use of temporary MCS is crucial [4,7,8,9].

The presented algorithm consists of five parts:

- Diagnosis and indication
- Evaluation of reversible causes of hemodynamic instability
- Evaluation of contraindications for temporary MCS (tMCS)
- Treatment of PCCS using tMCS
- De-escalation and weaning from tMCS.

## 3. Diagnosis and Indication

So far, there are no uniform diagnostic criteria or recommendations regarding the indication for the treatment of patients with PCCS. The current EACTS/ELSO/STS/AATS expert consensus gives recommendations on different tMCS modalities; however, it does not provide the complete treatment pathway [4]. Despite this, veno-arterial extracorporeal circulatory support systems are primarily used in acute settings [5]. The presented algorithm includes a structured approach based on readily available clinical parameters, allowing for an allocation to differentiated tMCS therapy depending on the underlying etiology and pathophysiology [6,10].

Depending on the clinical scenario, a distinction can be made between intraoperative and immediate postoperative circulatory failure starting in the intensive care unit (ICU) [11].

- Criteria for intraoperative CS: Failure to wean from CPB or inability to close the thorax and/or increasing lactate level and catecholamine requirement on CPB [4,11].
- Criteria for postoperative CS in the ICU: Increasing catecholamine requirement or/and hemodynamic instability despite high-dose inotropic support [11]. Objective quantification of the catecholamine requirement should be performed using the vasoactive inotropic score (VIS score), which takes into account the cumulative drug support of the circulation (inotropes and vasopressors) [12].

In addition to the VIS score, other parameters such as arterial lactate, cardiac index, central/mixed venous saturation, and systemic vascular resistance should be taken into account. Due to the potential influence of the index cardiac procedure or the underlining pathology itself, the relation to pre- and intraoperative values is particularly important in PCCS evaluation [6].

### Vasoactive Inotropic Score

The VIS is a useful tool for quantifying inotropic and vasopressor support and correlates with the severity of shock [12]. It is calculated using the following formula:

VIS = dobutamine* + 10 × milrinone* + 100 × epinephrine* + 100 × norepinephrine* + 10,000 × IU/kg/min vasopressin

* Dosage in µg/kg/min

In case of obesity, the calculation is based on the patient’s lean body weight [12].

A VIS > 30 indicates severe PCCS, in which case mechanical circulatory support should be considered [13].

## 4. Reversible Causes of Hemodynamic Instability

Before initiating tMCS therapy, reversible causes related to the underlying disease and/or surgery should be ruled out. In addition to the common differential diagnoses of CS, these include:

- Technical problems (ventilation, medication administration, measurement errors)
- (Tension) pneumothorax
- Bleeding
- Myocardial ischemia
- Iatrogenic dissection
- Pericardial tamponade
- Pulmonary embolism

## 5. Contraindications for Temporary MCS

Mechanical circulatory support is an invasive and resource-intensive treatment that can be associated with relevant complications [5]. For this reason, the decision to continue a therapy, but also to change the therapy goals towards palliative care, should be made in interdisciplinary consensus between the surgeon, anesthesiologist/intensive care physician, VAD surgeon, and nursing staff [6]. Our algorithm specifies the following parameters as indicators of a palliative approach:

- Signs of severe cerebral damage
- Critical comorbidities
- Malignancy with an anticipated life expectancy of less than 6 months
- Documented patient wishes/patient directives
- pH < 6.9 mol/L
- Lactate > 225 mg/dL (25 mmol/L) [14]
- No viable treatment options available
- Uncontrolled bleeding
- Ineffective resuscitation (MAP < 50 mmHg or apO2 < 50 mmHg for 30 min)

## 6. Temporary Mechanical Circulatory Support for PCCS Treatment

The selection algorithm for an optimal tMCS system is based on the clinical scenario and the severity of shock. The following points are taken into account when choosing the tMCS modality:

- Expected duration of support
- Anatomical characteristics of the patient (vascular access)
- Complication profiles of respective tMCS devices
- Availability of the tMCS devices

### 6.1. Isolated Left Ventricular (LV) Dysfunction

- In case of isolated LV dysfunction, without arrhythmia or severe lactic acidosis (<72 mg/dL or <8 mmol/L), primary implantation of an Impella pump (Abiomed, Danvers, MA, USA) should be performed [6,15].
- In patients with a mechanical aortic valve prosthesis or a free-floating thrombus in the LV, a left ventricular Impella is contraindicated. In such cases, primary implantation of v-a ECLS has to be performed. Following this, LV unloading strategies (e.g., IABP, transseptal percutaneous venting, direct LV venting via the LV apex or pulmonary artery) should be discussed.
- The Impella CP (Abiomed, Danvers, MA, USA) is usually implanted percutaneously via femoral artery and can generate a flow of up to 3.5 L/min. For patients with an expected short tMCS duration, sole percutaneous Impella CP implantation can be considered.
- The Impella 5.5 can provide up to 5.8 L/min depending on afterload and has to be surgically implanted through a vascular prosthesis. Axillary artery is the preferred access route, as it allows for early postoperative mobilization of the patient and uncomplicated explantation.
- If the axillary artery is less than 7-mm in diameter, exhibits calcifications or anatomical peculiarities (such as arteria lusoria), implantation of an Impella CP via the axillary artery, possibly on the contralateral side, may alternatively be considered.
- Alternative access routes such as the ascending aorta (in open-chest patients) can be considered.
- We recommend performing surgical implantation via a 10 (8)-mm vascular prosthesis, which is anastomosed in end-to-side fashion to the target vessel.

### 6.2. Isolated Right Ventricular (RV) Dysfunction

In case of isolated RV dysfunction, it is possible to establish RV support using v-a ECLS, a temporary right ventricular assist device (RVAD), or a right-sided percutaneous microaxial pump (Impella RP, Abiomed, Danvers, MA, USA) [5].

- In patients with an open chest, cannulation of the pulmonary artery can be performed using a vascular prosthesis (10- or 8-mm diameter), which is tunneled out of the chest cavity allowing for thoracic closure and facilitating later explantation. For venous drainage, a cannula is usually placed in the right atrium via femoral or the right internal jugular vein. Alternatively, if no peripheral access is possible and extended support duration is anticipated, direct cannulation of the right atrium can be performed. In this case, a 14 mm graft is usually anastamosed to right atrium and exteriorized and a 24–26 Fr venous cannula is directly inserted and chest is closed [9,16].
- In patients with a closed chest, percutaneous cannulation of the pulmonary artery can be performed via the right internal jugular vein. This can be achieved through two separate cannulas (long standard venous cannulas placed into the pulmonary artery and femoral vein), requiring two separate venous punctures [16].
- Alternatively, the insertion of a double-lumen cannula (ProtekDuo by LivaNova PLC, London, UK) facilitated right ventricular support through a single puncture of the jugular vein. The tip of the catheter is placed in the main stem or upper right pulmonary artery under fluoroscopic and echocardiographic guidance. This method allows for early mobilization on ongoing right ventricular support [17].
- The percutaneous Impella RP is inserted into the pulmonary artery via the femoral vein and generates a flow of up to 4.6 L/min. Disadvantages of the Impella RP are a limited support duration, impaired patient mobilization, and the lack of respiratory support [5]. Alternatively, the recently developed Impella RP Flex (Abiomed, Danvers, MA, USA) can be implanted through right internal jugular vein allowing mobilization of the patients.

### 6.3. Ongoing CPR

- Establishing rapid circulatory support is essential in patients undergoing CPR. In this case, immediate implantation of veno-arterial extracorporeal life support (v-a ECLS) is recommended [1,11,15].
- In open-chest patients, cannulation of central vessels (ascending aorta, right atrium) can be considered.
- In patients with a closed thorax, peripheral v-a ECLS cannulation should be performed. Whether percutaneous or open surgical cannulation is chosen depends on the clinical situation, anatomical circumstances of the patient, and the surgeon’s preference.
- In patients with peripheral arterial occlusive disease, surgical exposure of the femoral vessels and surgical implantation should be performed as a primary approach.
- Placement of a peripheral perfusion cannula is recommended, but can be performed shortly after hemodynamic stabilization.

### 6.4. Severe Cardiogenic Shock

In patients with biventricular heart failure, respiratory failure, and/or severe lactic acidosis (>72 mg/dL, >8 mmol/L), isolated Impella therapy cannot provide sufficient hemodynamic support. At the same time, sole support with v-a ECLS may lead to pulmonary congestion due to the afterload increase associated with the retrograde arterial flow of v-a ECLS [7]. Numerous studies have shown that LV unloading on v-a ECLS leads to a significant improvement in myocardial recovery potential and survival, with the effect seeming to depend on how fast LV unloading is achieved [4,7,8,9,17]. Recent data also suggest that prophylactic LV unloading is superior to a wait-and-see approach and with treatment performed if left ventricular distension already occurs [9]. Leading reviews and meta-analyses call for a consistent unloading therapy under v-a ECLS, which is why the algorithm presented here recommends sole v-a ECLS only in acute situations (especially ongoing CPR), complemented by LV unloading shortly afterwards [7,8]. This can be done in various ways:

#### 6.4.1. ECMELLA Approach

In our algorithm, we primarily recommend LV unloading for v-a ECLS using an Impella pump, the so-called ECMELLA (alternatively, ECPELLA) concept [6].

- ECMELLA combines the advantages of v-a ECLS and an Impella pump, namely: biventricular unloading through a simultaneous preload and afterload decrease, as well as pulmonary support with an oxygenator. Therapy on ECMELLA provides intensive mechanical circulatory support with high-volume flow, which is intended to ensure sufficient organ perfusion in phases of acute shock [7].
- Another important advantage of the ECMELLA concept is the easy way of de-escalation once circulatory conditions have been stabilized. In this case, v-a ECLS weaning is usually performed first while continuing Impella therapy. This enables longer support with a lower risk of complications [18].
- The ECMELLA 2.0/2.1 implantation technique represents the further development of the concept, whereby only one instead of two arterial cannulation sites is required to establish ECMELLA support [19,20].
- In this case, arterial vascular access is achieved via a Y-shaped vascular prosthesis, which is anastomosed to the axillary artery. One branch is used for Impella implantation, the other for the arterial cannula insertion of v-a ECLS. This method enables early postoperative mobilization (if venous drainage is performed via the right internal jugular vein) and bedside de-escalation on the ICU over time [19,20].

#### 6.4.2. Alternative Methods for Left Ventricular Unloading

*Intra-aortic balloon pump (IABP)*

The sole use of an IABP did not show any survival benefit in the treatment of cardiogenic shock patients [21].

- In our algorithm, the IABP is used only in combination with v-a ECLS for LV unloading in patients with absolute contraindications for an Impella pump. These include mechanical aortic valve prostheses or a floating thrombus in the left ventricle [6,15]. However, it should be noted that the degree of LV unloading achieved by an IABP depends strongly on the contractility of the left ventricle [22].

*Percutaneous venting*

Passive LV unloading can also be achieved with the help of a percutaneously placed cannula that is introduced into the left atrium by transseptal puncture [16].

- The venting cannula of the TandemHeart system (LivaNova PLC, London, UK) enables unloading of the left ventricle. It is connected to the venous drainage [16].
- The Bio-Medicus, NextGen two-stage cannula (Medtronic PLC, Dublin, Ireland) allows for simultaneous drainage of both atria [17].
- A percutaneous cannula is placed under fluoroscopy and echocardiographic control.
- In specific cases (e.g., pre-operated patients or complex vascular status), percutaneous atrial septostomy can be performed as a last-resort option.
- *Surgical venting*
- In patients with an open thorax an additional venous cannula can be placed through the upper right pulmonary vein into the LV [16].
- Alternatively, a vent can be placed directly via the LV apex. This method does not necessarily require a median sternotomy. Apical LV venting can be performed through a left lateral mini-thoracotomy, taking into account potential complications such as coronary artery injury, ventricular perforation, and bleeding [16].
- After placement, the cannulas for passive venting are tunneled outwards, fixated, and connected to the venous drainage of the v-a ECLS using a Y-shaped connector.

## 7. De-Escalation and Weaning from tMCS

**Figure 2:**
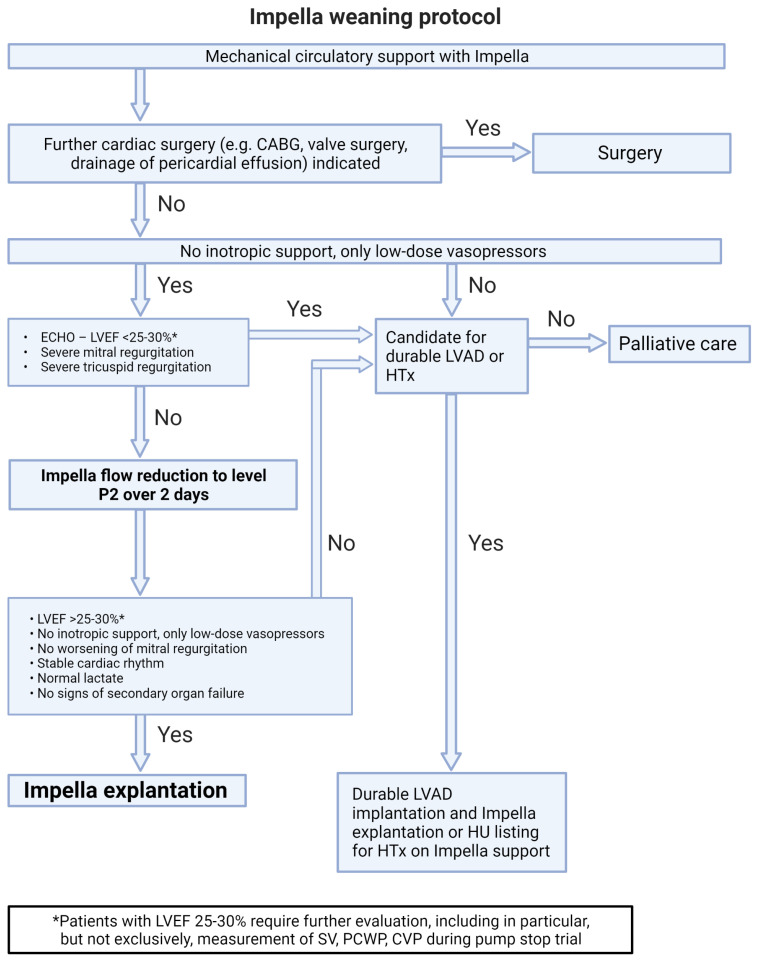
Impella weaning protocol. Abbreviations: CABG—coronary artery bypass grafting; LVEF—left ventricular ejection fraction; HTx—heart transplantation; HU—high urgency, SV—stroke volume; PCWP—pulmonary capillary wedge pressure; CVP—central venous pressure.

**Figure 3:**
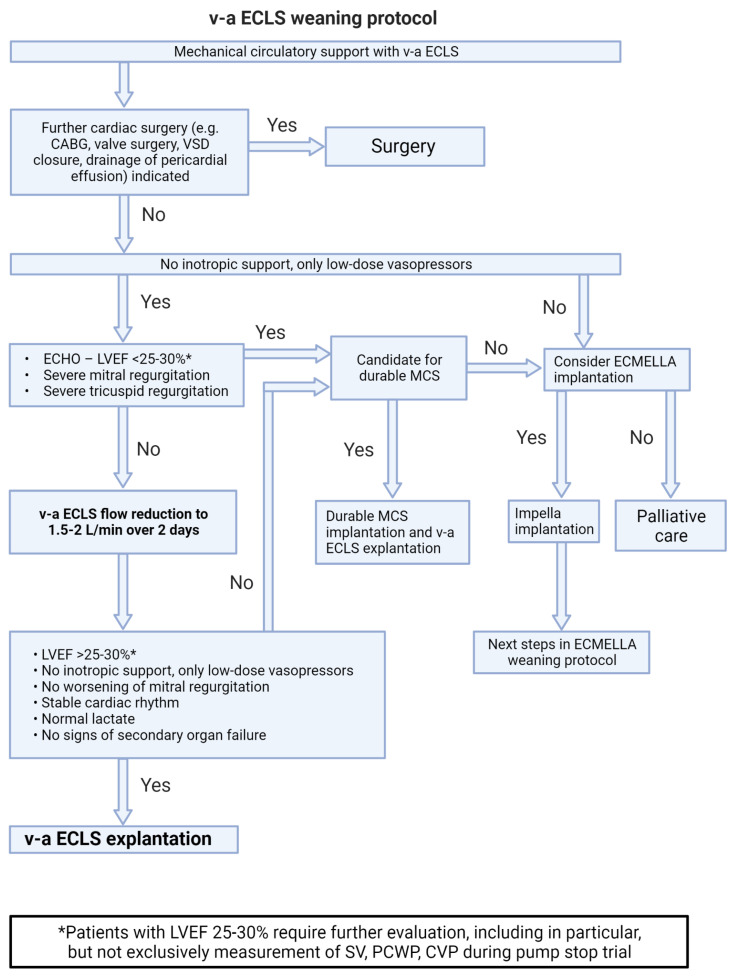
v-a ECLS weaning protocol. Abbreviations: v-a ECLS—veno-arterial extracorporeal life support; CABG—coronary artery bypass grafting; LVEF—left ventricular ejection fraction; VSD—ventricular septal defect; MCS—mechanical circulatory support; SV—stroke volume; PCWP—pulmonary capillary wedge pressure; CVP—central venous pressure.

**Figure 4:**
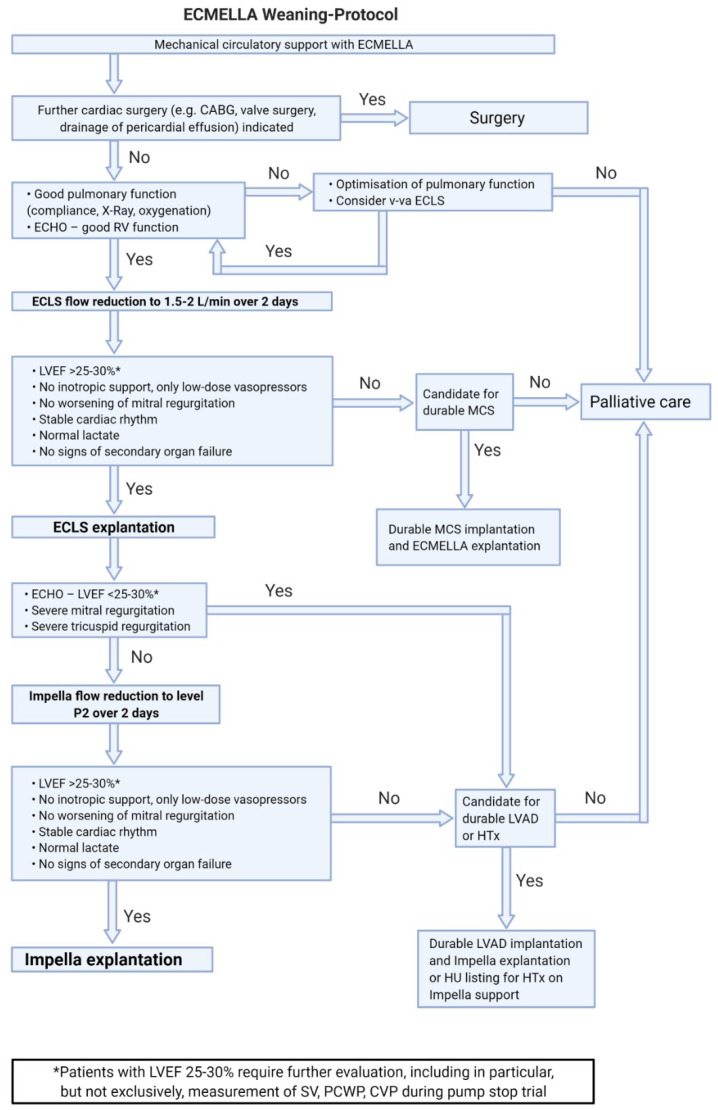
ECMELLA weaning protocol. Abbreviations: v-a ECLS—veno-arterial extracorporeal life support; CABG—coronary artery bypass grafting; LVEF—left ventricular ejection fraction; LVAD—left ventricular assist device; HTx—heart transplantation; HU—high urgency; MCS—mechanical circulatory support; SV—stroke volume; PCWP—pulmonary capillary wedge pressure; CVP—central venous pressure.

**Figure 5:**
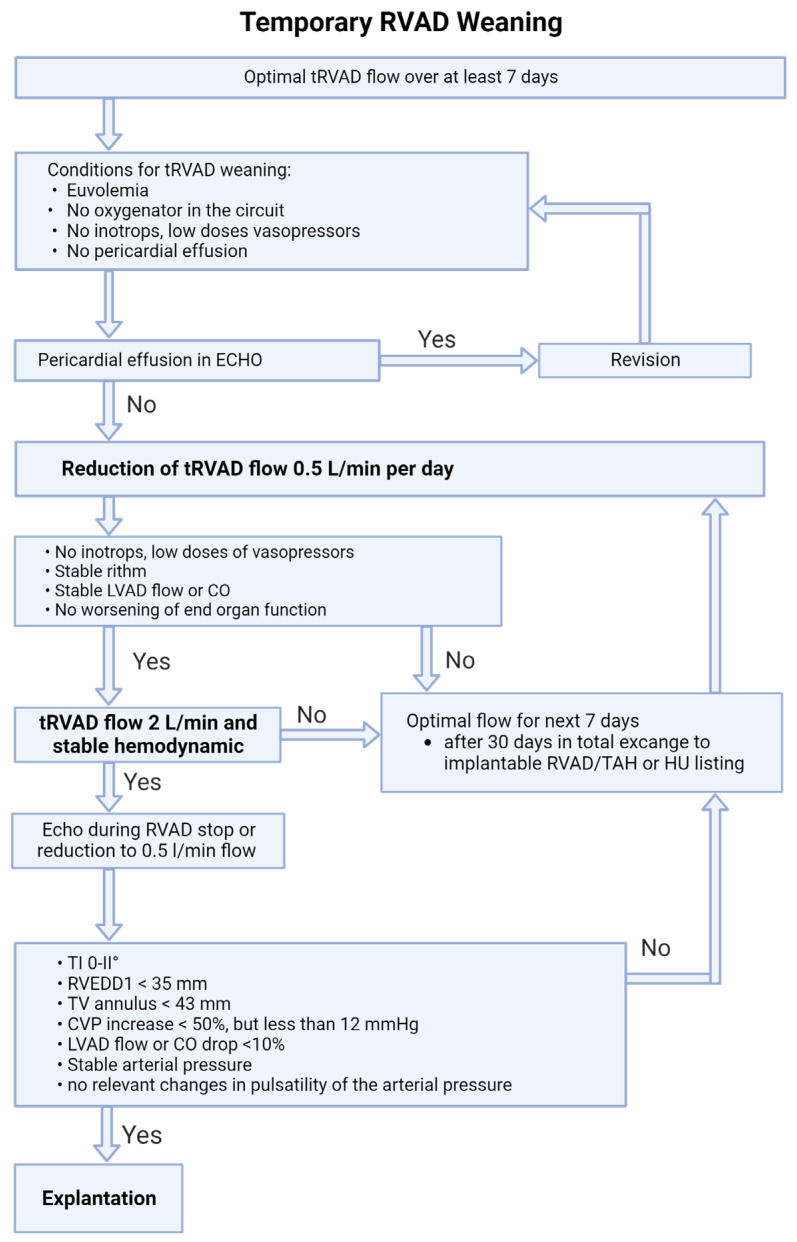
RVAD weaning protocol. Abbreviations: tRVAD—temporary right ventricular assist device; CO—cardiac output; LVAD—left ventricular assist device; RVEDD—right ventricular enddiastolic diameter; TV—tricuspid valve; TI—tricuspid insufficiency; CVP—central venous pressure.

Systems for tMCS represent an effective therapy for PCCS but carry the risk of treatment-associated complications. Those probability increases with the duration of support; therefore, a daily evaluation of the weaning potential is of great importance. Depending on the tMCS systems used, Figure 2, Figure 3, Figure 4 and Figure 5 represent algorithms for weaning and de-escalation from temporary mechanical circulatory support.

First, the indication for re-operation or intervention is verified depending on the entity of the cardiogenic shock (e.g., bypass/ coronary artery occlusion, pericardial effusion, severe valve dysfunction).

The basic prerequisites for weaning are the cessation of inotropic support (low-dose vasopressors are allowed) and a LVEF > 30% (measured at minimal circulatory support level). If the LVEF is <25%, long-term support system should be considered. In borderline cases with an LVEF of 25–30%, a detailed re-evaluation of cardiac function should be performed after optimization of heart failure therapy [6,10].

### 7.1. Discontinuation of Isolated Left Ventricular Impella Support (Figure 2)

- After hemodynamic stabilization and a significant reduction of inotropic medication (with only low-dose vasopressor therapy remaining), a stepwise reduction in support is performed until the P2 level is achieved. This is done gradually with a continuous re-evaluation of hemodynamics over at least 48 h. Support should not be reduced below the P2 level, as this can cause retrograde flow through the pump into the left ventricle.
- If there are no severe valve pathologies, the LVEF is at least 25–30%, there is a stable rhythm, and no continuous inotropic treatment is needed while on support at the P2 level, the Impella can be removed.
- If this is not the case, alternative treatment concepts such as LVAD, heart transplantation, or palliation should be discussed.
- In case of increasing mitral regurgitation during a reduction of Impella flow, endovascular mitral valve reconstruction can be performed under Impella support [23].
- If circulatory support is inadequate (increasing catecholamine demand and increasing arterial lactate despite maximum Impella therapy), v-a ECLS implantation for escalation to ECMELLA can be considered, but long-term LVAD therapy should be prioritized [6].
- In the event of severe respiratory failure, veno-venous extracorporeal membrane oxygenation (v-v ECMO) can be implanted.
- If severe hemolysis, pump thrombosis, or Impella pump failure occur, a switch to a new Impella can be performed, but long-term LVAD therapy should be simultaneously discussed depending on the cardiac recovery potential.

### 7.2. Weaning from v-a ECLS Support (Figure 3)

- The basic criteria for weaning from v-a ECLS are the same as for weaning from Impella support. After stabilization of the patient’s hemodynamic condition, the v-a ECLS flow is gradually reduced to 1.5–2 L over at least 48 h.
- If the general weaning criteria (no higher-grade valve pathologies, LVEF of at least 25–30%, stable rhythm, no inotropes) are met, v-a ECLS can be explanted.
- In patients on ECLS with unclear neurological status or severe complications, switch to Impella support in setting of bridge-to-decision therapy should be considered [24].
- Harlequin syndrome (also known as differential hypoxemia) is a rare complication that can occur after onset of the unloading device or during myocardial recovery under v-a ECLS therapy. In severe respiratory failure, poorly oxygenated blood is ejected into the circulation, but remains in the supra-aortic vessels due to retrograde flow and a high afterload generated by v-a ECLS. In this situation, the therapy should be escalated to veno-veno-arterial ECLS. To do this, an additional arterial ECLS line is established in order to transport oxygenated blood to the right atrium. This counteracts differential hypoxemia and reduces the risk of cerebral and coronary hypoperfusion [25].

### 7.3. Weaning from ECMELLA Support (Figure 4)

- The basic criteria for ECMELLA weaning correspond to those for v-a ECLS and Impella weaning. Complications on v-a ECLS are common and increase with support duration. Therefore, the concept of ECMELLA weaning focuses primarily on the reduction of v-a ECLS support with potential explantation.

- ECMELLA therapy with femoral cannulation and simultaneous respiratory insufficiency also bears a risk of differential hypoxemia (Harlequin syndrome). In this case, escalating to vv-a ECMELLA should be considered.
- If de-escalation of ECMELLA therapy is not possible, long-term LVAD implantation on ECMELLA can be performed.

### 7.4. Weaning from Temporary RVAD Support (Figure 5)

The right ventricle has a significant regenerative potential, but sometimes requires prolonged periods of support. Therefore, implantation of a permanent RVAD or heart transplantation should be considered only after longer periods of support (>30 days) [5]. It should also be considered that implantation of a permanent RVAD is a complex off-label procedure with a significant potential for complications [26].

- We recommend a stepwise flow reduction of the temporary RVAD by no more than 0.5 L/min per day until 2 L/min is reached.
- It is important to underline, that in case of temporary RV support with Impella RP device the flow should not be reduced below 1.5 L/min (P4 level), due to the iatrogenic tricuspid regurgitation caused by the device itself.
- If hemodynamics fail to stabilize at minimal flow, the optimal support level should be established and re-evaluation performed after seven days.
- If weaning from temporary RVAD support is not possible after a total of 30 days, switching to a permanent system and/or listing for heart transplantation should be considered [10].
- If gas exchange is impaired during temporary RVAD support, the installation of an oxygenator in the extracorporeal circuit should be considered.

## 8. Discussion

Despite advances in therapy, the survival of patients with CS after cardiac surgery has hardly improved in recent years [2]. In contrast to CS in the setting of acute myocardial infarction or acute decompensated chronic heart failure, the technical aspects of the surgery play a central role. The allocation algorithm presented here aims to standardize the therapy decision-making process for critical CS patients and minimize delays in establishing adequate support.

The protocols are based on current studies and expert recommendations in the field of mechanical circulatory support. In a retrospective analysis, Ott et al. evaluated the results of the first version of the institutional allocation protocol for selecting temporary MCS in a large cardiac surgery department. A propensity score-matched analysis demonstrated a significant improvement in 30-day survival in patients treated according to the protocol (56.9% vs. 38.9%, *p* = 0.044) [10].

LV unloading for CS patients treated with v-a ECLS is a crucial component of the presented algorithm, as it is associated with a significant survival benefit [7,8]. However, it should be noted that combined tMCS therapy in the form of an ECMELLA concept leads to a significant increase in complications, such as bleeding, hemolysis, and limb ischemia [7]. Although mortality is still significantly lower, the rationale for preventive unloading in v-a ECLS-treated patients with a sufficient output remains questionable. Radakovic et al. found that patients who received prophylactic LV unloading via Impella during v-a ECLS therapy, independent of LV dilation, showed a significantly better survival and a higher rate of left ventricular recovery [9].

In this current manuscript, we summarized the most recent recommendations on tMCS therapy and adapted them for use in the setting of PCCS. We also presented de-escalation strategies for various tMCS modalities that can be applied to common causes of cardiogenic shock. However, our expert group remains committed to continuously improving and optimizing the presented tMCS algorithms.

## 9. Conclusions

Cardiogenic shock after cardiac surgery is a life-threatening condition associated with high mortality. Tailored therapy using temporary mechanical circulatory support is an effective and often life-saving treatment option for patients with PCCS. The algorithm-based standardization of tMCS treatment seems a useful strategy to optimize the time-sensitive care of patients with PCCS.
